# Host age and expression of genes involved in red blood cell invasion in *Plasmodium falciparum* field isolates

**DOI:** 10.1038/s41598-017-05025-5

**Published:** 2017-07-05

**Authors:** Aida Valmaseda, Quique Bassat, Pedro Aide, Pau Cisteró, Alfons Jiménez, Aina Casellas, Sonia Machevo, Ruth Aguilar, Betuel Sigaúque, Virander S. Chauhan, Christine Langer, James Beeson, Chetan Chitnis, Pedro L. Alonso, Deepak Gaur, Alfredo Mayor

**Affiliations:** 1 ISGlobal, Barcelona Ctr. Int. Health Res. (CRESIB), Hospital Clínic - Universitat de Barcelona, Barcelona, Spain; 2 Centro de Investigação em Saúde de Manhiça (CISM), Manhiça, Mozambique; 3 ICREA, Pg. Lluís Companys 23, 08010 Barcelona, Spain; 4 Centro de Investigación Biomédica en Red de Epidemiología y Salud Pública (CIBEREsp), Madrid, Spain; 5 Laboratory of Malaria and Vaccine Research, School of Biotechnology, Jawaharlal Nehru University, New Delhi, India; 6 Malaria Group, International Centre for Genetic Engineering and Biotechnology (ICGEB), New Delhi, India; 7 Macfarlane Burnet Institute for Medical Research and Public Health, Melbourne, Victoria Australia

## Abstract

*Plasmodium falciparum* proteins involved in erythrocyte invasion are main targets of acquired immunity and important vaccine candidates. We hypothesized that anti-parasite immunity acquired upon exposure would limit invasion-related gene (IRG) expression and affect the clinical impact of the infection. 11 IRG transcript levels were measured in *P. falciparum* isolates by RT-PCR, and IgG/IgM against invasion ligands by Luminex®, in 50 Mozambican adults, 25 children with severe malaria (SM) and 25 with uncomplicated malaria (UM). IRG expression differences among groups and associations between IRG expression and clinical/immunologic parameters were assessed. IRG expression diversity was higher in parasites infecting children than adults (p = 0.022). *eba140* and *ptramp* expression decreased with age (p = 0.003 and 0.007, respectively) whereas *p41* expression increased (p = 0.022). *pfrh5* reduction in expression was abrupt early in life. Parasite density decreased with increasing *pfrh5* expression (p < 0.001) and, only in children, parasite density increased with *p41* expression (p = 0.007), and decreased with *eba175* (p = 0.013). Antibody responses and IRG expression were not associated. In conclusion, IRG expression is associated with age and parasite density, but not with specific antibody responses in the acute phase of infection. Our results confirm the importance of multi-antigen vaccines development to avoid parasite immune escape when tested in malaria-exposed individuals.

## Introduction

By invading host cells, apicomplexan parasites can develop in a rich nutrient environment while being protected from host defenses. In *Plasmodium falciparum*, invasion of red blood cells (RBC) by merozoites is mainly driven by the cooperative function of ligands from the *P. falciparum* reticulocyte-binding homolog (PfRh) and the erythrocyte-binding antigen (EBA) families^1–3^. In addition, many ligands that do not belong to these families have been identified as having key roles during RBC invasion^4–6^. The expression of invasion-related genes (IRG) during schizont stages^7^ is tightly regulated as part of the parasite life cycle^8, 9^. Depending on the nature of the RBC receptor used, the invasion phenotype can be classified as sialic acid (SA)-dependent or SA-independent^10^. Some invasion ligands have been linked to each of these two phenotypes in culture-adapted parasite lines namely PfRh1, EBA175, and EBA140 to SA-dependent pathway^11^ and PfRh2a, PfRh2b and PfRh4 to SA-independent pathway^12–17^. Inverse correlations in expression between genes involved in opposite pathways (*pfrh1* vs. *pfrh2b*) have been observed in natural isolates from Kenya^18^ and Tanzania^19^. However, it was observed that the same invasion phenotype does not always involve the same gene expression pattern^8^.

The essential process of RBC invasion is extremely rapid^20^, probably for minimizing the exposure of merozoites to host-derived antibodies targeting invasion ligands. This immune recognition can lead to inhibition of the parasite replication or killing and therefore constitute a selection pressure on the parasite population^5^. Antibodies to a range of merozoite invasion ligands are acquired through exposure to malaria and have been associated with protection from clinical illness^21^. In return, the parasite has developed immune evasion strategies through the high polymorphism^22^, functional redundancy or gene variant expression of critical invasion ligands^8, 13, 23^. Indeed, the parasites that survive during an infection are thought to express variants corresponding to gaps in the repertoire of host antibodies^24^, and variation in invasion ligand use by the parasite during RBC invasion has been shown to mediate evasion of inhibitory antibodies^25, 26^. Consequently, this immune pressure may modulate IRG expression, as it is hypothesized for *eba175* ^27, 28^, and changes in EBA175 expression and/or use by the parasite alter the susceptibility to acquired inhibitory antibodies^25^. Interestingly, few studies have reported that particular transcripts of IRG correlate with parasitemia, age or endemicity levels^28, 29^. Moreover, differences have been reported in multiplication rates and invasiveness in natural isolates from Thai patients with severe (SM) and uncomplicated (UM) malaria^30, 31^. However, no differences have been found between SM and UM patients in other parasite populations regarding multiplication rates, RBC selectivity, invasion phenotypes^32, 33^ or IRG expression levels^18, 28^.

The immune escape of parasites caused by exposure to vaccine-induced anti-malarial immunity can be minimized if the IRGs whose expression is modulated by immune pressure are identified^34^. Therefore, this kind of studies can help to adapt vaccination strategies based on the predicted change in malaria transmission intensity (and therefore in accumulated exposure) caused by malaria control and elimination campaigns. Here we aimed to study the gene expression levels of IRGs in *P. falciparum* isolates collected from Mozambican individuals with different levels of previous exposure to malaria parasites, defined by their age or their antibody responses against *P. falciparum* antigens. We hypothesized that anti-parasite immunity acquired after years of exposure would limit the expression of IRG by the infecting parasites and determine the clinical impact of the infection. To address this, we compared IRGs expression in parasites infecting adults and children from the same area and studied the association with their immune status and clinical outcomes.

## Materials and Methods

### Study area and population

The study was conducted in the Manhiça District, southern Mozambique^35^, where a demographic surveillance system (DSS) set up in 1998 by the Centro de Investigação em Saúde de Manhiça (CISM), currently provides accurate demographic information on its *circa* 178,000 inhabitants. The region has a warm and rainy season from November to April, and a cooler and drier season the rest of the year. Malaria transmission is perennial with some seasonality. *Plasmodium falciparum* is the predominant species, and *Anopheles funestus* the main vector. Entomological inoculation rate in 2002 was 38 infective bites/person/year^35^.

Patients attending the Manhiça District Hospital with a clinical diagnosis of *P. falciparum* malaria were recruited into the study after obtaining written informed consent. Samples were collected between April-November 2006 (children; n = 25 SM patients and n = 25 UM patients) and August 2006 to May 2008 (adults, n = 50, UM). Clinical malaria was defined as the presence of fever (axillary temperature ≥ 37.5 °C) with an asexual parasitemia of *P. falciparum* ≥ 500/μL on thin blood film examination. SM was defined when children had, at least, one of the following symptoms: cerebral malaria (Blantyre Coma Score ≤ 2), severe anemia (packed cell volume < 15% or hemoglobin < 5 g/dL), acute respiratory distress (lactate > 5 mM and/or chest indrawing or deep breathing), prostration, hypoglycemia (blood glucose < 2,2 mM) and multiple seizures ( ≥ 2 convulsions in the preceding 24 h)^36^. Individuals with UM were those with clinical malaria not showing any of the mentioned signs of severity. Parasite density was determined by optic microscopy. Peripheral blood was collected by venipuncture before treatment. Following centrifugation, the RBC pellet was cryopreserved in liquid nitrogen^36^. The studies were approved by the Mozambican National Bioethics Committee and the Hospital Clínic of Barcelona Ethics Review Committee and all experiments were carried out in accordance with relevant guidelines and regulations.

### Parasite culture and quantification of transcript levels

Cryopreserved *P. falciparum* isolates were thawed and matured as previously described^36^. Cultures were monitored by Giemsa-stained thin smears for 18–56 hours until most schizont stages were observed, as determined by the agreement of two trained researchers. Infected RBCs were collected, washed with PBS, mixed with 20 V of TRIzol® and RNA was obtained following manual extraction with phenol/chloroform phase separation. Total RNA was treated with Turbo DNA-*free*™ kit (Ambion) at 37 °C for 45 minutes and reverse transcribed with oligo(dT) using Maxima H reagents (Thermo scientific).

Quantitative PCR (qPCR) was performed on an ABI PRISM-7500 real-time system (Applied Biosystems) using 2μL of cDNA in a final volume of 20μL, including 10μL of Power SYBR green master mix (Applied Biosystems) and primers for specific genes at the convenient concentration (see Supplementary Table 1). Gene-specific primers were designed to match conserved regions only in *P. falciparum* based on the coding sequence (GeneBank) and rechecked with sequences available in www.plasmodb.org on October 2015. Human DNA was not amplified with any primer. To obtain transcript concentration from each sample, the mean Ct (duplicates) of the sample was interpolated to a 7-point 10-log standard curve with 3D7 genomic DNA of known concentrations, for each of the studied genes. Samples with a Ct > 30 for the housekeeping gene (Seryl tRNA synthetase) were discarded, and samples with a Ct higher than the higher Ct from each gene’s standard curve, were considered as not expressed. Each IRG transcript level was normalized as a proportion of the sum of all transcripts concentrations in each isolate and reported as a percentage of expression (relative transcript levels). Expression was considered only if relative transcript level was higher than 0. Multiplicity of infection in children was measured as described elsewhere^37^.

### Antibody measurement

Apical asparagine-rich protein (AARP), Plasmodium Thrombospondin-related apical merozoite protein (PTRAMP), EBA175PfF2, PfRh1, PfRh2a/b, PfRh4_30_, PfRh4_C-terminal_, PfRh5, P41 and Cysteine-rich protective antigen (CyRPA) were produced at the International Centre for Genetic Engineering and Biotechnology (ICGEB)^38–42^. PfRh2_2030–2528_ and regions III–V of EBA175 (EBA175_III–V_) and EBA140 (EBA140_III–V_) were produced at the Burnet Institute^26, 43, 44^. Recombinant proteins were expressed in *Escherichia coli*. IgG and IgM responses targeting *P. falciparum* antigens were measured by quantitative suspension array technology (qSAT) in multiplex using xMAP™ beads (Luminex Corporation) coupled to each antigen. Positive, negative and background controls were added to each plate. Multiplexed beads were incubated with plasma samples, and antibody levels were measured as described elsewhere^45, 46^. Median Fluorescence Intensity (MFI) was obtained from the InVitrogen Luminex® platform (xPONENT® Software, at least 100 counts/analyte) and normalized for inter-plate variability by multiplying individual values by the median value of a positive control assayed in all plates and dividing by each plate’s value. Negative values were substituted by the minimum positive value divided by two in order to log-transform these variables.

### Definitions and statistical analysis

Age was categorized as < 2.5 years; 2.5 years to ≤ 5; 14 to < 22 years and 22 to ≤ 61 years according to quartile distribution. Seropositive threshold was defined as the mean plus 3 standard deviations of the value of the negative population given by finite mixture models estimated with log-transformed antibody levels. Breadth of antibody responses was defined as the sum, for each individual, of the number of specific antibodies above the seropositive threshold. The ratio for genes from the SA-dependent/SA-independent pathways was obtained from *eba175, eba140* and *pfrh1* for SA-dependent and from *pfrh2a, pfrh2b* and *pfrh4* for SA-independent pathways. The diversity of gene expression in each *P. falciparum* isolate was defined as the number of genes with transcripts detected by qPCR.

To compare mean levels or prevalence between groups, we used Student’s t-test (or Wilcoxon rank-sum test if the variable was not normally distributed) and chi-square test, respectively. Spearman’s correlations were calculated to assess strength of the relationship between IRG levels and parasite density. Comparisons between more than two groups were done with Kruskal-Wallis test for non-normally distributed data. To assess the association between variables, we performed crude and adjusted linear regression models, including interaction terms with age. Wald’s test was used to assess the overall effect of age on relative transcript levels. Reported p-values were not adjusted for multiple comparisons^21, 47^ and therefore, the interpretation of results is based on the standard significance level (p < 0.05) and on the magnitude of the effect. Statistical analyses were performed using Stata (StataCorp. 2015. Stata Statistical Software: Release 14. College Station, TX: StataCorp LP) and graphs with Prism7 (GraphPad).

## Results

The 100 clinical *Plasmodium falciparum* isolates thawed for *ex vivo* culture developed and reached late trophozoite/schizont stages. Three of them (3%) were discarded due to a Ct value > 30 for the housekeeping gene (Seryl tRNA synthetase). All plasma samples from adults and children were tested for specific IgG and IgM levels against the recombinant proteins produced from the studied genes (except for 1 adult and IgM determination in 8 children with UM and 4 children with SM due to unavailability of enough plasma volume). Out of the 96 samples analyzed for transcript and IgG antibody levels, 46 were adults (48%) and 50 children (52%). No statistically significant differences in terms of parasitemia, gender or parasites’ maturation time between groups were found (Table 1).

**Table 1:** Demographic, clinical and parasitological factors of the infected individuals included in this study.

	Adults	Children			P-value	
		**All**	**UM**	**SM**	**UM-SM**	**Adults vs Children**
**N**	46	50	25	25		
**Age (years), median (IQR)**	24 (18, 34)	2.6 (1.3, 3.5)	2.6 (1.1, 3.3)	2.8 (1.6, 3.6)	0.327^a^	< 0.0001^a^
**Sex (male % (n))**	47.8% (22)	60% (30)	68% (17)	52% (13)	0.248^b^	0.232^b^
**Parasitemia (OM, parasites/uL), median (IQR)**	38654.5 (25924, 82880)	47659.6 (22927.8, 81632.7)	42989.9 (19109.2, 73714.3)	50402.4 (23327.55, 114372.5)	0.327^a^	0.760^a^
**Maturation time (hours at 37 °C), median (IQR)**	31.3 (28, 39)	32 (30, 39)	35.5 (31, 39)	32 (30, 38)	0.219^a^	0.863^a^

^a^Wilcoxon rank-sum test; ^b^Chi-square test. Abbreviations: UM, Uncomplicated malaria; SM, Severe malaria; IQR, Interquartilic range; OM, Optic Microscopy.

### Expression of invasion-related genes and specific antibody levels

The most abundant transcripts in the studied isolates were from *p41*, with median relative transcript levels of 15.2% (interquartile range [IQR] 6.4%, 29.5%), followed by *eba140* (12.6% [5.6%, 22.1%]) and *ptramp* (7.6% [5.7%, 10.7%]), followed by *cyrpa* (6.1% [4.4%, 10.7%])*, eba175* (5.9% [0.2%, 14.2%]) and *aarp* (4.5% [9%, 12%]). Genes from the *pfrh* family were the least expressed (see Supplementary Table 2). No consistent correlation was found for relative transcript levels of genes involved in the SA-dependent pathway (*eba175, eba140, pfrh1*) or the SA-independent pathway (*pfrh2a, pfrh2b, pfrh4)*, nor among them (see Supplementary Table 3). No differences were found in the ratio for relative transcript levels reflecting SA-dependent or SA-independent pathways among adults, children with SM and children with UM (Kruskal-Wallis test, p-value = 0.735). Relative transcript levels did not differ in infections with different levels of multiclonality (Kruskal-Wallis test, all p-values > 0.05).

### Relative transcript levels of invasion-related genes, by age and specific antibody levels

**Figure 1:**
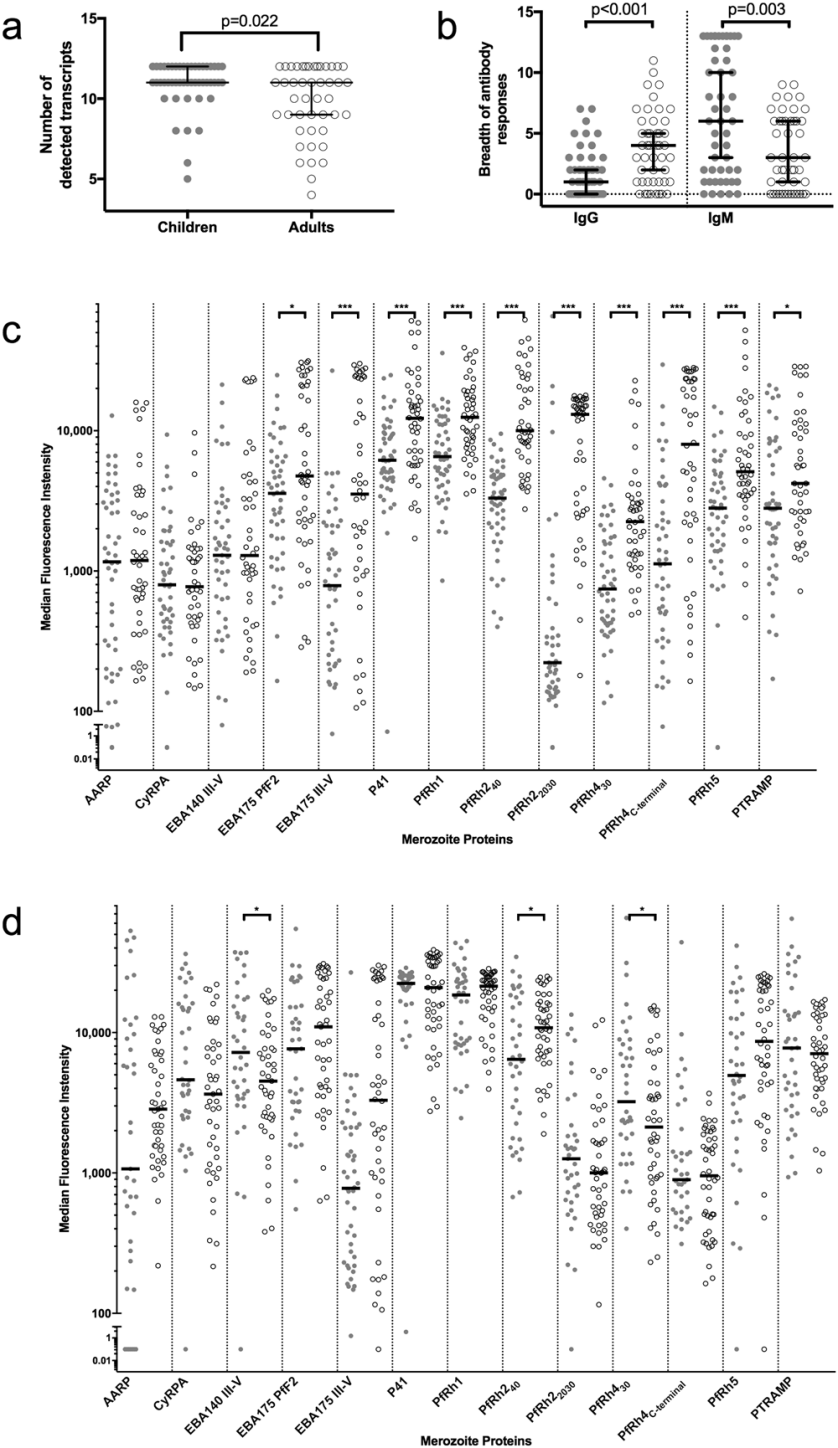
Diversity of invasion-related gene expression, breadth of antibody antibody responses and specific antibody levels against invasion-related antigens in Mozambican adults and children. (**a**) Diversity of invasion-related genes expression in each age group. Children had a higher diversity compared to adults (11 and 10, respectively, Wilcoxon rank-sum test, p = 0.022). Median values with the 95% confidence intervals are shown. (**b**) Breadth of antibody responses of specific IgG and IgM responses in age groups. Adults have higher breadth of IgG antibody responses against malaria antigens than children (Wilcoxon rank-sum test, p < 0.001) whereas children have higher breadth of IgM antibody responses (Wilcoxon rank-sum test, p = 0.003). Median values with the 95% confidence intervals are shown. (**c**,**d**) Specific IgG (**c**) and IgM (**d**) levels in adults and children. *p < 0.05; **p < 0.01; ***p < 0.001. Grey dots represent values for children and empty dots values for adults. Median values are represented by horizontal black lines.

The median of different transcripts detected within the parasite isolates studied was 10 (9, 12). This median was higher in children (11 [11, 12]) compared to adults (10 [9, 12]) (p = 0.022, Fig. 1a). Diversity in IRG expression was independent of parasite density (Spearman’s rho = 0.07, p = 0.497). Breadth of IgG antibody responses was higher in adults (p < 0.001) and, mirroring the higher diversity in IRG expression in children than in adults, children also showed higher breadth in IgM antibody responses than adults (p = 0.0027, Fig. 1b). No association between parasite density and IgG or IgM breadth was observed (p > 0.05).

Levels of IgG antibodies against the antigens studied were, in general, higher in adults than children: EBA175 PfF2 (p = 0.036), EBA175_III–V_ (p < 0.001), P41 (p < 0.001), PfRh1 (p < 0.001), PfRh2_40_ (p < 0.001), PfRh2_2030_ (p < 0.001), PfRh4_30_ (p < 0.001), PfRh4_C-terminal_ (p < 0.001), PfRh5 (p < 0.001) and PTRAMP (p = 0.039). IgM levels were higher in children for antibodies against PfRh4_30_ (p = 0.047), PfRh2_40_ (p = 0.039) and EBA140 _III–V_ (p = 0.021). Specific IgG and IgM antibody levels are showed in Fig. 1c,d, respectively. No differences in antibody levels were found between children with SM or UM, except for IgM against P41 (p = 0.008) and IgM against CyRPA (p = 0.037), both being higher in UM compared to SM patients.

**Figure 2:**
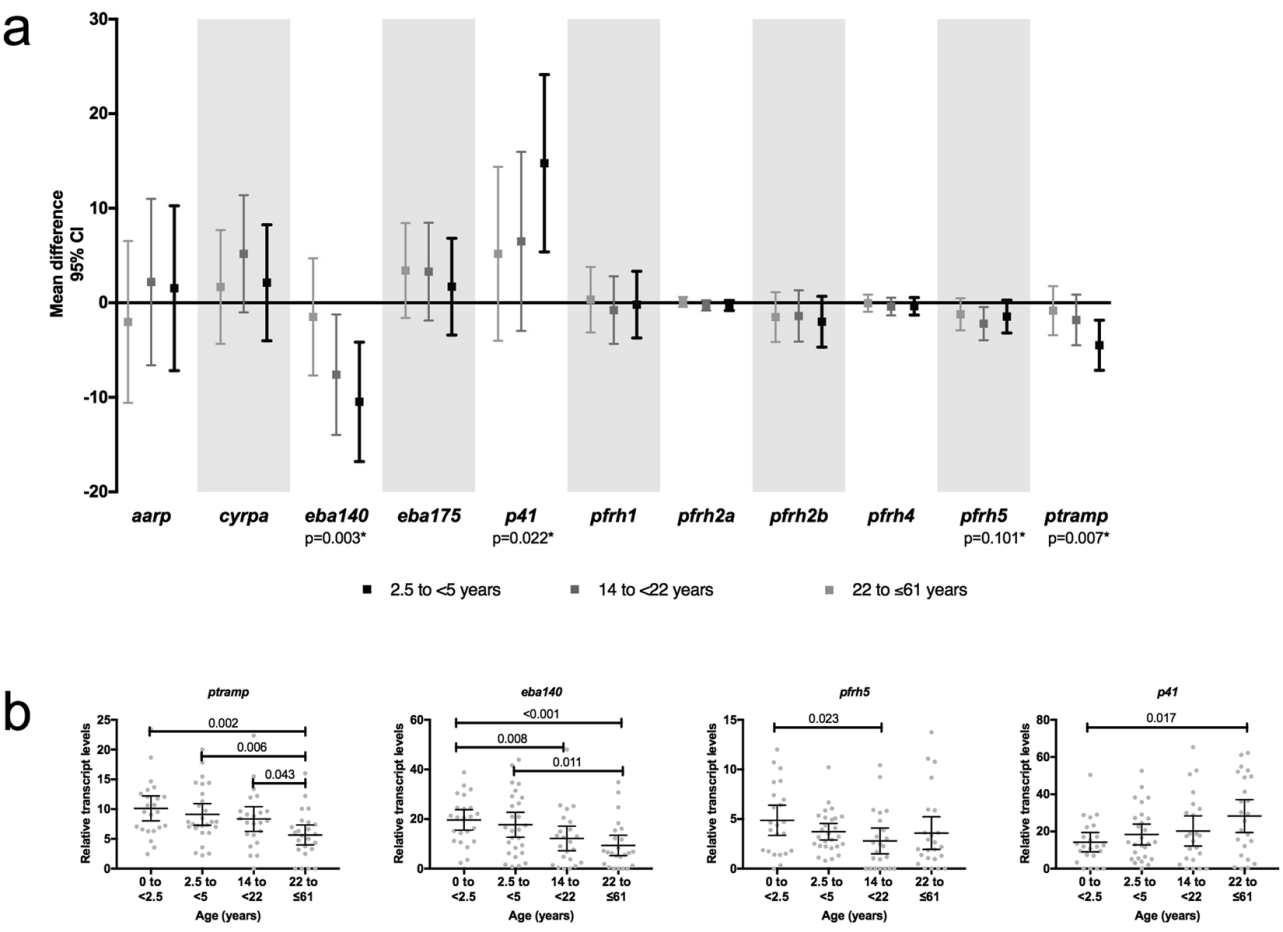
Association of age with invasion-related gene relative transcript levels. (**a**) Mean difference between age groups and 95% confidence interval from linear regression models, adjusted by density of infection, where the reference group was the youngest children (less than 2.5 years of age). *P-values from Wald’s test for significant associations of any age group with IRG relative transcript levels. (**b**) Relative transcript levels of IRG with significant associations with age by age groups. Significant differences between age groups by Wilcoxon rank-sum test are specified.

**Figure 3:**
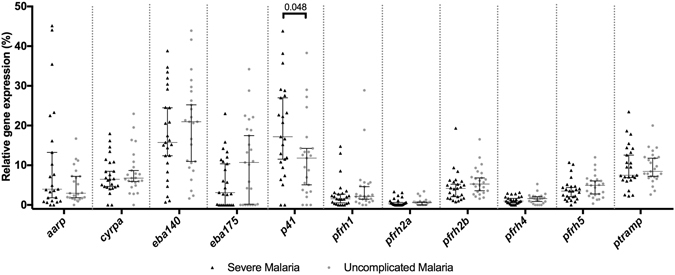
Relative transcript level of invasion-related genes in children with severe and uncomplicated malaria. Transcript levels are shown for individual isolates and median values with the 95% confidence interval for each group; significant differences are specified (Wilcoxon rank-sum test, p < 0.05). IRG relative transcript levels of each *P. falciparum* isolate is represented with black triangles for parasites from severe malaria patients and with grey circles for parasites from uncomplicated malaria patients.

Relative transcript levels of *eba140* and *ptramp* were lower in older than in younger individuals (p = 0.003 and p = 0.007, respectively), while *p41* relative transcript levels increased with age (p = 0.022) (Fig. 2a). The expression of *eba140, ptramp* and *p41* by infecting parasites showed a gradual change as the age of the host increased. In contrast, *pfrh5* relative transcript levels were higher in children than in adults (Wilcoxon rank-sum test, p = 0.013), and decreased abruptly during firsts years of life and reached the lowest levels in parasite infecting young adults (Wilcoxon rank-sum test, p = 0.023, Fig. 2b). Among children, *p41* was more expressed in parasite isolates from children with SM (p = 0.048) than with UM (Fig. 3). This difference was slightly higher when only considering those samples with *p41* transcripts detected (p = 0.043). Interestingly, higher levels of relative transcript levels for *eba175* in parasites causing UM compared to SM was only found when considering parasites with *eba175* transcripts detected (p = 0.03).

No consistent relationships between relative transcript levels and specific antibodies (IgG and IgM) against the recombinant merozoite antigens were found (Supplementary Table 4). Despite IgG breadth being higher in adults than in children and IgM breadth higher in children than in adults, no general association was found between breadth of IgG or IgM antibody responses and IRG relative transcript levels (Supplementary Figure 1a,b, respectively). Interaction with age was only significant for *ptramp* relative transcript levels and IgG breadth. The association between IgG breadth and *ptramp* relative transcript levels was only significant in children (Coefficient −0.93 [95% confidence interval −0.29, −1.70]; p = 0.007). Therefore, data presented in Supplementary Figure 1 was not stratified.

### Transcript levels of invasion-related genes are associated with parasite density

**Figure 4:**
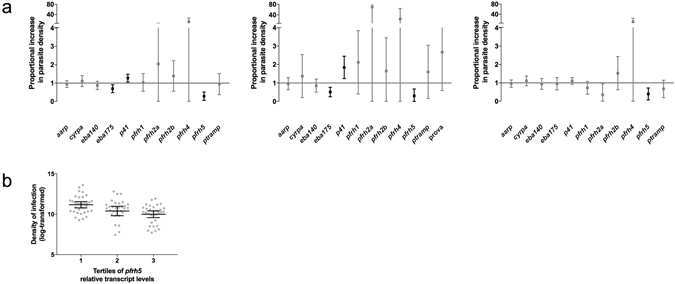
Association of invasion-related genes (IRG) relative transcript levels and parasite density. (**a**) Effects of 10% increase in relative transcript levels on parasite density by optic microscopy (linear regression models adjusted by age and clinical presentation) are shown with the 95% confidence interval for each specific IRG. Black symbols represent significant associations between IRG relative transcript levels and parasite density. From left to right: all population, only children and only adults. (**b**) Parasite density by optic microscopy according to tertile categorization of *pfrh5* relative transcript levels in the parasite population.

Parasite density had a proportional increase of 1.3 (95% CI 1.1, 1.5; p = 0.007) with a 10% increase in *p41* relative transcript levels. In contrast, parasite density decreased with increasing levels of *eba175* (Coef. 0.7 [95% CI 0.5, 0.9]; p = 0.013) and *pfrh5* (Coef. 0.2 [95% CI 0.1, 0.5]; p < 0.001), (Fig. 4a). The relationship of *eba175* and *p41* relative transcript levels with parasite density was affected by the fact of being an adult (age groups from 14 to < 22 and 22 to ≤ 61 years old) or children (age groups from 0 to < 2.5 and 2.5 to < 5 years old) (p-values of interaction = 0.021 and 0.005, respectively). After stratifying the analysis, it was observed that in children, but not in adults, parasite density was positively associated with *p41* relative transcript levels (Coef. 1.8 [95% CI 1.3, 2.5]; p = 0.001) and negatively associated with *eba175* (Coef. 0.5 [95% CI 0.3, 0.8]; p = 0.005) (Fig. 4a). In contrast, the reduction of parasite density with increasing relative transcript levels of *pfrh5* was similar in children (Coef. 0.2 [95% CI 0.03, 0.7]; p = 0.02) and adults (Coef. 0.3 [95% CI 0.1, 0.8]; p = 0.01), (Fig. 4a). Parasite density for each tertile of *pfrh5* relative transcript levels is shown in Fig. 4b.

## Discussion

This is, to our knowledge, the first study to analyze the expression of a wide array of *P. falciparum* genes involved in invasion among parasite isolates collected from Mozambican individuals covering a broad range of ages, from early infancy to late adulthood. Such an approach allowed us to identify invasion-related genes (IRG) whose relative transcript levels vary depending on the age of the infected host. The relative transcript levels of *eba140*, *ptramp* and *pfrh5* were the highest in parasites infecting young individuals, while *p41* relative transcript levels were the highest in older individuals. This points out how different histories of previous exposure can modulate the expression of genes involved in RBC invasion, probably due to the development of antimalarial immunity upon exposure to the parasite.

The diversity of IRG expression was higher in children than in adults. Such diversity was not associated with parasite density and therefore a sensitivity issue due to cDNA available during the qPCR procedure, which could bias our results, can be discarded. Thus, it is shown that older individuals have a higher ability to limit the diversity of IRG transcripts during an infection compared to children^24^, and presumably select the parasites expressing a reduced IRG repertoire. Among the specific IRG analyzed, *eba140*, *ptramp* and *pfrh5* were found to be more expressed in children than in adults, while *p41* was less expressed in children than in adults. The decrease in diversity of IRG expression with age is also probably due to the decrease in relative transcript levels of the IRG described here (except for *p41*). No associations were found between specific antibody levels and IRG relative transcript levels. This may be because antibodies were measured at the acute phase of infection, when an overall boosting of immune responses is generally observed^48^. Alternatively, levels of antibodies may not accurately reflect their functional inhibitory activity^26, 49, 50^ and therefore not fully representing the selective pressure of immunity on ligand expression. In addition, the role of other immune effectors or a global immune action (including humoral and cellular responses) cannot be discarded, as well as the possibility of a general reset of the epigenetic machinery during transmission stages^51^, which in early phases of infection would restart IRG expression. This expression might then be shaped in the first rounds of invasion depending on the host’s antimalarial immunity.

The reduction of the relative transcript levels of *eba140*, *ptramp* and *pfrh5* with age can be due to the pressure exerted by the acquired anti-malarial immunity, which increases with age in our study population. It is possible that these invasion ligands may be naturally expressed at moderate to high levels in the absence of a selective pressure^24^. During an infection, this would turn into a very low-restricted expression of these IRG upon exposure to immune effectors. This change in expression levels would be even more rapid when the ligand targeted is highly conserved and/or essential for the parasite survival^42, 52^, such as *pfrh5*, as we have observed. This can explain the consistent association described in this study between high relative transcript levels of *pfrh5* and lower parasite densities, contrary to what was previously described^28, 29^: the more *pfrh5* expressed, the more exposure to the immune system and therefore higher selective pressure in favor of those parasites expressing less *pfrh5* ^53^. This observation is consistent with the epidemiological pattern where infection densities decrease with increasing age in malaria endemic areas^26^. However, it cannot be discarded that the differences observed with age are due to factors other than anti-malarial immunity developed upon exposure, such as maturity or the immune system itself^ 54, 55^.

In this study, *p41* relative expression was positively associated with age and, only in children, with higher parasite densities. In this population, expression of *p41* was slightly higher in parasites infecting children with SM, than UM (although very borderline significance). We also report higher IgM levels against P41 in children with UM compared to children with SM, maybe reflecting a protective role of IgMs against severe malaria. All together suggest that *p41* might be important in the success of the invasion process and affected by anti-parasite host immunity. However, the mechanisms remain still unknown, since *p41* is the only gene studied here that has not been directly linked to invasion^56^. More studies will be useful to both determine the role of *p41* in the parasite’s biology as well as the effect of specific antibodies in protection against malaria severity.

In the case of *eba175*, its expression levels were slightly higher in children with UM compared to SM, although it did not reach statistical significance. This is in accordance with previous results^28^ showing that invasion by parasites causing UM had the higher dependence on trypsin (and a trend to neuraminidase-sensitive phenotype). Interestingly, isolates from The Gambia showed that SA-dependent invasion is more common among infections in children^57^. Also, although it is not always linked to SA-dependent pathway^58^, it has been hypothesized that *eba175* can be more expressed in non-immune individuals^28^. In addition, the acquisition of inhibitory antibodies against antigens from the SA-dependent pathway may be acquired early in life^25^. Here, we show that IgG levels against antigens from the SA-independent pathway (PfRh2 and PfRh4, Fig. 1c) are much higher in adults than in children; early antibody responses (IgM) against PfRh4 are lower in adults; and that expression of the SA-dependent pathway antigen *eba140* is lower in adults. Taken together, these evidences suggest the possibility that what is being selected in parasites infecting immune individuals is the invasion phenotype rather than invasion ligands themselves, although further studies are needed.

This study has some limitations that need to be considered. First, we cannot discard that differences in the relative transcript levels between parasites collected from children and adults are due to different duration of the infections, which could affect IRG expression levels. Second, the maturation process of multi-clonal infections could lead to the selection of those clones with higher maturation rates. Given that there is no reference gene to normalize for maturation stage, transcript levels were expressed as relative to the sum of all genes studied to control for temporal transcript patterns during cell cycle development, as has been done in previous studies^27, 28^. Third, there may be a lack of concordance between transcript and protein levels for the merozoite proteins expressed by field isolates^18, 19^. Also, it has not been possible to study the invasion phenotype, which would have helped to clarify its relationship with previous malaria exposure. Finally, the possibility of type I errors derived from analyses reporting non-adjusted p-values should be taken into account^47^, and, although the overall results of our study suggest that expression of genes is modulated by factors modified by age, other studies would be required to confirm this statement.

In conclusion, this study shows that the expression of invasion ligands used by *P. falciparum* to invade RBCs can be modulated by the age of the infected host, possibly as a surrogate of immunity. These results suggest that the efficacy of malaria vaccines aiming to block the invasion process may differ between individuals with different levels of malaria exposure. Also, the observation that children (or non-immune individuals) are infected by parasites expressing a more diverse IRG repertoire, supports the potential need of combining several RBC binding ligands in a multi-antigenic vaccine to avoid that the parasite escapes the immune responses induced by single-target vaccines. Finally, results reported here suggest that the loss of immunity associated with reductions in malaria transmission after elimination or control campaigns, could lead to a change in the repertoire of IRG expressed by the remaining parasite population, and therefore to changes in the efficacy of invasion-blocking vaccines.

## Electronic supplementary material

Supplementary Material

Supplementary Dataset 1
