# Supplementary material for: Host age and expression of genes involved in red blood cell invasion in Plasmodium falciparum field isolates
Source: Sci Rep. 2017 Jul 5;7:4717. doi: 10.1038/s41598-017-05025-5 (PMC5498679; doi:10.1038/s41598-017-05025-5)
Supplement: Supplementary file 1 — Supplementary Material [file 41598_2017_5025_MOESM1_ESM.docx]

**Host age and expression of genes involved in red blood cell invasion in *Plasmodium falciparum* field isolates**

Aida Valmaseda^1*^, Quique Bassat^1,2,3^, Pedro Aide^2^, Pau Cisteró^1^, Alfons Jiménez^1,4^, Aina Casellas^1^, Sonia Machevo^2^, Ruth Aguilar^1^, Betuel Sigaúque^2^, Virander S. Chauhan^6^, Christine Langer^7^, James Beeson^7^, Chetan Chitnis^6^, Pedro L Alonso^1,2^, Deepak Gaur^5^, Alfredo Mayor^1,2^^[[1]](#footnote-1)^

^1^ ISGlobal, Barcelona Ctr. Int. Health Res. (CRESIB), Hospital Clínic - Universitat de Barcelona, Barcelona, Spain. ^2^ Centro de Investigação em Saude de Manhiça (CISM), Moçambique. ^3^ ICREA, Pg. Lluís Companys 23, 08010 Barcelona, Spain. ^4^Centro de Investigación Biomédica en Red de Epidemiología y Salud Pública (CIBEREsp), Madrid, Spain. ^5^ Laboratory of Malaria and Vaccine Research, School of Biotechnology, Jawaharlal Nehru University, New Delhi, India. ^6^ Malaria Group, International Centre for Genetic Engineering and Biotechnology (ICGEB), New Delhi, India. ^7^ Macfarlane Burnet Institute for Medical Research and Public Health, Melbourne, Victoria, Australia.

# Supplementary tables legends

**Supplementary table 1**. Sequences and concentrations of primers for each of the genes studied by quantitative PCR. Fw: Forward; Rv: Reverse.

**Supplementary table 2.** Ranking of the relative transcript levels of invasion-related genes in the whole population (all) under study or stratified by age (children or adults). Median and interquartilic range are specified.

**Supplementary table 3. Correlations among genes involved in different invasion pathways (SA dependent or SA-independent)**. Spearman’s test was performed and significant p-values are shown in bold.

**Supplementary table 4. Association of specific IgG and IgM against invasion-related proteins on the relative transcript levels of IRG**. Coefficients of linear regression models with their 95% confidence interval are shown for unadjusted models or adjusted by the age, parasite density and study. Significant associations (p-value < 0.05) are shown in bold.

# Supplementary figure legend

**Supplementary figure 1. Association between breadth of antibody responses and invasion-related genes relative transcript levels.** Linear regression coefficients (models adjusted by age, parasite density and clinical presentation) are shown with the 95% confidence interval for each specific transcript for breadth of IgG **(a)** or IgM **(b)**. Coefficients indicate the difference in IRG relative transcript levels per additional antigen above seropositivivty threshold (one unit increase in breadth).

# Supplementary tables

**Supplementary table 1.**

| **Gene** | **Reference** | **Concentration** | **Gene ID (Genebank)** | **Sequences** |
| --- | --- | --- | --- | --- |
| *aarp* | [57] | 2µM | 24529620 | Fw: 5’-AACGAATGAAGAAGAGGAAGG-3’  Rv: 5’-TCTCATACTTAAATCAATAAAGGAACC-3’ |
| *cyrpa* | Self-design | 2µM | 812432 | Fw: 5’-TGACAATTACAAATTAGGTGTGCAA-3’  Rv: 5’-TCATCATTTAATGTAGAAACATCTTGA-3’ |
| *eba140* | Self-design | 2µM | 813949 | Fw: 5’-AGGAAAGTGCGGGGAATAGT-3’  Rv: 5’-CTAGAACAAGGACCCGGTGA-3’ |
| *eba175* | Self-design | 2µM | 2654998 | Fw: 5’-AACGGAAACTCGTACGGATG-3’  Rv: 5’-GTTTGGAATGGCGTCAGATT-3’ |
| *p41* | Self-design | 2µM | 812447 | Fw: 5’-GCGAATCCTTCGGATGATATT-3’  Rv: 5’-TTGTATTGTCACCGTGTCCAG-3’ |
| *pfrh1* | Self-design | 2µM | 9221810 | Fw: 5’-GAAATTCGCTCGGGACAATA-3’  Rv: 5’-TTGAATCAAGGGGGACAATTA-3’ |
| *pfrh2a* | Self-design | 2µM | 813730 | Fw: 5’-TTCTTATAACATAAAATGCTTGGTTTT-3’  Rv: 5’-AAGCACAATTTGTTTTCGAATG-3’ |
| *pfRh2b* | [18] | 2µM | 813730 | Fw: 5’-ACAGAAAGCGATGATATTGATAACAGTGAA-3’  Rv: 5’-CCCATGGGTGTTACTTCTATGACT-3’ |
| *pfrh4* | Self-design | 4µM | 812435 | Fw: 5’-TGGGTTACGACAAATGGAAA-3’  Rv: 5’-TGTGCATTATCCCAAAGGTG-3’ |
| *pfrh5* | [28] | 2µM | 812437 | Fw: 5’-ACGAAGAATCAAGAAAATAATCTGACGTTACT-3’  Rv: 5’-TGTTGAATGATCTTTAGCATTATTTGTTTTTATATTCTCTTT-3’ |
| *ptramp* | Self-design | 2µM | 811226 | Fw: 5’-GCGAAGAGGAGAAGGAGGAG-3’  Rv: 5’-AGGGAGAATTGTGTCCAATCA-3’ |
| *seryl* *tRNA*  *synthetase* | [58] | 2µM | 2655117 | Fw: 5’-AAGTAGCAGGTCATCGTGGTT-3’  Rv: 5’-TTCGGCACATTCTTCCATAA-3’ |

**Supplementary table 2.**

| Rank | All | Children | Adults |
| --- | --- | --- | --- |
| 1 | *p41*  15.4% (6%; 29.7%) | *eba140*  17% (10.6%; 26.3%) | *p41*  18.8% (5.5%; 41.1%) |
| 2 | *eba140*  12.7% (5.4%; 23.1%) | *p41*  13.1% (6.4%; 24.6%) | *eba140*  7% (1.9%; 15.8%) |
| 3 | *ptramp*  7.5% (5.6%; 11%) | *ptramp*  8.2% (6.8%; 12.6%) | *ptramp*  6.5% (3.7%; 9.4%) |
| 4 | *cyrpa*  6.1% (4.4%; 10.8%) | *cyrpa*  6.5% (4.6%; 10.7%) | *eba175*  5.9% (0.8%; 15.1%) |
| 5 | *eba175*  5.8% (0.2%; 14.1%) | *pfrh2b*  4.6% (2.6%; 6.4%) | *cyrpa*  5.7% (3.2%; 11.5%) |
| 6 | *aarp*  4.5% (1.9%; 11.8%) | *eba175*  5.8% (0%; 13.9%) | *aarp*  4.9% (2.2%; 15.3%) |
| 7 | *pfrh2b*  3.7% (1.7%; 6.2%) | *aarp*  3.7% (1.6%; 10.3%) | *pfrh2b*  2.6% (1.1%; 5.3%) |
| 8 | *pfrh5*  2.9% (1.4%; 5.3%) | *pfrh5*  3.7% (2.1%; 5.9%) | *pfrh5*  2.1% (0.5%; 3.9%) |
| 9 | *pfrh1*  1.5% (0.4%; 3.5%) | *pfrh1*  2% (0.7%; 3.5%) | *pfrh1*  0.9% (0%; 3.5%) |
| 10 | *pfrh4*  0.7% (0.1%; 1.8%) | *pfrh4*  1.3% (0.5%; 2.1%) | *pfrh4*  0.5% (0%; 0.8%) |
| 11 | *pfrh2a*  0.1% (0%; 0.7%) | *pfrh2a*  0.4% (0%; 0.8%) | *pfrh2a*  0.02% (0%; 0.4%) |

**Supplementary table 3.**

| Transcripts pair | | Spearman's rank rho | p-value |
| --- | --- | --- | --- |
| *eba175* | *eba140* | **0.263** | **0.009** |
| *eba175* | *pfrh1* | 0.037 | 0.716 |
| *eba175* | *pfrh2a* | 0.051 | 0.620 |
| *eba175* | *pfrh2b* | 0.072 | 0.484 |
| *eba175* | *pfrh4* | -0.003 | 0.977 |
| *eba140* | *pfrh1* | 0.157 | 0.123 |
| *eba140* | *pfrh2a* | **0.228** | **0.024** |
| *eba140* | *pfrh2b* | **0.327** | **0.001** |
| *eba140* | *pfrh4* | **0.236** | **0.020** |
| *pfrh1* | *pfrh2a* | 0.124 | 0.226 |
| *pfrh1* | *pfrh2b* | 0.047 | 0.649 |
| *pfrh1* | *pfrh4* | **0.324** | **0.001** |
| *pfrh2a* | *pfrh2b* | **0.236** | **0.020** |
| *pfrh2a* | *pfrh4* | 0.051 | 0.618 |
| *pfrh2b* | *pfrh4* | **0.204** | **0.045** |

**Supplementary table 4.**

|  |  | Unadjusted | | Adjusted by parasite density, age and study | |
| --- | --- | --- | --- | --- | --- |
| Invasion-related gene | Protein | IgG | IgM | IgG | IgM |
| *aarp* | AARP | 0.55 (-1.24; 2.35) | -0.49 (-1.65; 0.68) | 0.48 (-1.24; 2.20) | -0.59 (-1.75; 0.57) |
| *ptramp* | PTRAMP | -0.72 (-1.60; 0.166) | 0.68 (-0.48; 1.85) | -0.67 (-1.55; 0.21) | 0.31 (-0.86; 1.48) |
| *cyrpa* | CyRPA | -1.15 (-2.77; 0.48) | -0.94 (-2.40; 0.52) | -1.23 (-2.86; 0.39) | -0.95 (-2.44; 0.54) |
| *ama1* | AMA1 | -0.49 (-2.25; 1.28) | 1.39 (-0.57; 3.35) | -0.72 (-2.79; 1.35) | 1.42 (-0.64; 3.47) |
| *p41* | P41 | 1.75 (-1.40; 4.89) | -0.76 (-4.28; 2.77) | 0.89 (-2.07; 3.85) | 0.27 (-2.94; 3.48) |
| *eba140* | EBA140 IIIV | -0.65 (-2.27; 0.97) | -0.02 (-1.66; 1.61) | -0.37 (-1.92; 1.17) | -0.61 (-2.13; 0.91) |
| *eba175* | EBA175 PfF2 | 0.77 (-0.66; 2.21) | 1.02 (-0.75; 2.80) | 0.27 (-1.25; 1.79) | 0.49 (-1.40; 2.38) |
|  | EBA175 IIIV | 0.42 (-0.57; 1.40) | 0.69 (-0.42; 1.80) | 0.04 (-1.08; 1.16) | 0.49 (-0.65; 1.63) |
| *pfrh1* | PfRh1 | -0.30 (-1.20; 1.39) | -0.35 (-2.20; 1.50) | -0.29 (-2.22; 1.62) | -0.48 (-2.47; 1.50) |
| *pfrh2a* | PfRh2_40_ | **-0.18 (-0.35; -0.01)** | -1.23 (-0.32; 0.07) | -0.17 (-0.41; 0.07) | -0.14 (-0.36; 0.07) |
|  | PfRh2_2030_ | -0.04 (-0.12; 0.04) | 0.03 (-0.09; 0.16) | -0.01 (-0.11; 0.09) | 0.03 (-0.10; 0.15) |
| *pfrh2b* | PfRh2_40_ | -0.52 (-0.39; 0.36) | 0.30 (-0.79; 1.39) | -0.36 (-1.58; 0.85) | 0.18 (-1.01; 1.38) |
|  | PfRh2_2030_ | 0.02 (-0.38; 0.42) | 0.15 (-0.56; 0.85) | 0.20 (-0.30; 0.70) | 0.07 (-0.65; 0.79) |
| *pfrh4* | PfRh4_30_ | -0.15 (-0.46; 0.16) | -0.05 (-0.25; 0.35) | -0.05 (-0.42; 0.33) | 0.04 (-0.29; 0.36) |
|  | PfRh4_C-terminal_ | 0.06 (-0.12; 0.25) | 0.05 (-0.28; 0.39) | 0.16 (-0.05; 0.37) | 0.04 (-0.30; 0.38) |
| *pfrh5* | PfRh5 | 0.32 (-0.13; 0.76) | 0.11 (-0.22; 0.44) | **0.46 (0.03; 0.90)** | 0.05 (-0.27; 0.38) |

# Supplementary figures

**Supplementary figure 1.**

1. **Corresponding author**: Dr. Alfredo Mayor, ISGlobal, Barcelona Centre for International Health Research, Hospital Clínic – Universitat de Barcelona, Carrer Rosselló 153 (CEK Building), E-08036 Barcelona, Spain. Telephone +34 93 227 5400 – ext 4519. E-mail: [alfredo.mayor@isglobal.org](mailto:alfredo.mayor@isglobal.org); **Alternative corresponding author**: Aida Valmaseda, ISGlobal, Barcelona Centre for International Health Research, Hospital Clínic – Universitat de Barcelona, Carrer Rosselló 153 (CEK Building), E-08036 Barcelona, Spain. Telephone +34 93 227 5400 – ext 3388. Email: [aidavalmaseda@gmail.com](mailto:aidavalmaseda@gmail.com)alfredo.mayor@isglobal.org [↑](#footnote-ref-1)
